# Microbiological contamination of lettuce (*Lactuca sativa*) reared with tilapia in aquaponic systems and use of bacillus strains as probiotics to prevent diseases: A systematic review

**DOI:** 10.1371/journal.pone.0313022

**Published:** 2024-11-11

**Authors:** Angélica Adiação Jossefa, Leonildo dos Anjo Viagem, Brunno da Silva Cerozi, Sebastian Wilson Chenyambuga

**Affiliations:** 1 Departamente of Animal, Aquaculture and Range Sciences, Sokoine University of Agriculture, Morogoro, Tanzania; 2 Higher School of Rural Development, Eduardo Mondlane University, Inhambane, Mozambique; 3 Department of Food and Agricultural, Rovuma University, Morogoro, Cabo Delgado, Mozambique; 4 Department of Animal Science, College of Agriculture, University of Sao Paulo, Piracicaba, São Paulo, Brazil; Universidad Autonoma de Chihuahua, MEXICO

## Abstract

Aquaponic systems are food production systems that combine aquaculture and hydroponic in a closed recirculation system where water provides nutrients to plants while plants purify water for fish. In this system, tilapia is the most commonly cultured fish and can be easily integrated with vegetable cultivation. However, tilapia host a diverse microbiota some of which are pathogenic and can infect humans. Previous studies have reported contamination of lettuce by pathogenic bacteria which can cause human diseases. Thus, there is an urgent need to employ effective methods to control those bacteria, and Bacillus strains have been successfully used in this context. This systematic review aimed to provide a comprehensive overview of lettuce contamination by pathogenic bacteria and the use of Bacillus as probiotics to prevent diseases in aquaponics systems. This systematic review was performed using Preferred Reporting Items for Systematic Review and Meta-Analysis Statement (PRISMA) Guidelines. A total of 1,239 articles were retrieved and based on eligibility criteria, six articles were included after screening. The review revealed that *Enterobacteriaceae*, *Coliforms*, and *Shiga Toxin-producing E*. *coli* are the predominant bacteria contaminating lettuce leaves in Aquaponic systems, and *Shiga Toxin-Producing E*. *coli* can internalize in the lettuce leaves, putting public health at risk. The included studies did not report the presence of *V*. *cholerae* in lettuce grown in aquaponic systems, and the use of Bacillus as probiotics to control *Escherichia coli* and *Vibrio Cholerae*. Further research is needed to explore the potential of tilapia to act as a source of pathogenic bacteria that can contaminate lettuce, as well as to investigate the effectiveness of Bacillus strains as probiotics to control these bacteria and ensure food safety.

## Introduction

Aquaculture is experiencing rapid growth compared to other food production sectors and playing a vital role in ensuring food security [1–4]. Despite its benefits, the expansion of aquaculture is negatively impacting aquatic ecosystems [5]. This has led to the widespread adoption of environmentally sustainable systems such as aquaponic systems around the world [6, 7]. In aquaponic systems, vegetables are grown alongside tilapia in a closed recirculation system where water is not discarded [8], and nutrients from fish tanks are used as a source of plant nutrition [6]. According to [9], food safety is compromised when vegetables are grown in the presence of fish farming effluent.

Human illnesses and deaths caused by the consumption of food contaminated with pathogenic bacteria are a constant threat to public health worldwide [10, 11]. *Escherichia coli* and *Vibrio cholerae* are considered highly pathogenic to humans and have been identified as parts of the microbiota of tilapias [12–14]. Lettuce is the most common raw food associated with foodborne illnesses globally [15], and is also the most commonly cultivated in aquaponic systems [8]. Tilapia, which is the most commonly cultured fish in aquaponic systems [11], can harbor a diverse microbiota [12–14], some of which are pathogenic to humans and can be transmission through the consumption of uncooked food [16, 17].

In recent years, the use of aquaponic systems for food production has raised concerns about the potential contamination of vegetables by microorganisms [18]. Several researchers [16, 19–23], have recommended conducting further studies on the potential contamination of vegetables grown in aquaponic systems, highlighting the multiple pathways through which pathogenic bacteria can be introduced. These researchers have emphasized the need for research to understand how and when microbial contamination occurs in aquaponic systems, driven by concerns over food safety. They also proposed investigating preventive strategies to reduce the risk of contamination of vegetables grown in aquaponic systems.

Due to the risks of microbiological contamination in aquaponic systems, it is recommended to adopt preventive methods to control diseases [21]. The use of probiotics may be a safe alternative [24] as they do not pose a risk to the health of cultured fish and vegetables or humans [21]. In aquaponic systems, probiotics can be introduced either through feed supplementation or direct inoculation into the water, and they can be employed either as single-species preparation or as a combination of multiple species [25]. *Bacillus* spp. is one of the most commonly used probiotic bacteria in aquaculture [26]. Specifically, *B*. *subtilis* and *B*. *licheniformis* have been shown to improve growth performance and reduce pathogens in Nile tilapia [27]. According to [28], *B*. *subtilis* can be mixed with malic acid to reduce pathogens in the gut of Nile tilapia, and *B*. *licheniformis* can be combined with other probiotics and give good results in the host.

This systematic review aimed to comprehensively outline the existing research on lettuce contamination by pathogenic bacteria in aquaponic systems, specifically *E*. *coli* and *V*. *cholerae*. Several studies [29–37] have reported the presence of these pathogens in tilapia intestines and rearing water. According to these studies, *V*. *cholerae* is a significant human pathogen that can proliferate in aquatic environments and spread to the human intestine. This bacterium can colonize the tilapia intestine, and survive for at least 15 days. *E*. *coli* is one of the most prevalent microorganisms in food, with various strains that differ in their pathogenicity on the host. Outbreaks caused by these two pathogens are often associated with the consumption of uncooked food, which can become contaminated through waterborne feces. The internalization of these pathogens in lettuce leaves can put public health at risk. Therefore, their presence and prevention in aquaponic systems should be investigated to ensure food security. We also aimed to elucidate the efficacy of Bacillus strains in controlling these pathogens in aquaponic systems, as these probiotics have been extensively used in aquaculture and have shown the potential to control pathogenic bacteria.

## Methods

This systematic review followed the Preferred Reporting Items for Systematic Review and Meta-Analysis (PRISMA) Statement 2015, PRISMA 2020 Checklist (S1 Checklist), and the protocol (S1 Protocol) was registered on Open Science Framework (OSF) with the associated project: osf.io/va3nk and registration Doi: https://doi.org/10.17605/OSF.IO/K64EN.

### Database source

A search was conducted in September 2023 across three databases: Google Scholar, Science Direct, and Wiley online library, for studies published between 2013 and 2023. The search focused on articles discussing microbial contamination of lettuce and the use of *B*. *subtilis* and *B*. *licheniformis* as probiotics to control *E*. *coli* and *V*. *cholerae* in aquaponic systems.

### Search strategy

The following search was used to find relevant articles in the three databases: 1. “microbial” AND “contamination” AND “lettuce” AND “Aquaponic system”; 2. “lettuce” AND “*Bacillus subtilis*” AND “*E*. *coli” OR “Vibrio cholerae” AND “aquaponic systems”;* 3. “lettuce” AND *“Bacillus licheniformis”* AND “*E*. *coli” OR “V*. *cholerae” AND “Aquaponic system”*. Additionally, *relevant references cited in the selected studies*, *but not found in the databases using the above search terms*, *were manually searched and cross-checked to include them in the study*, *based on the eligibility criteria*.

### Eligibility criteria

#### Inclusion criteria

Retrieved articles had to meet the following criteria to be included in the study: **i**. published between 2013 and 2023; **ii.** written in English; **iii.** available in full text; **iv**. contain original research that combines tilapia and lettuce farming in aquaponic systems; **v.** Articles where tilapia was challenged with *E*. *coli* or *V*. *cholerae*; **iv.** focus on *B*. *subtilis* and *B*. *licheniformis* as a probiotic to control *E*. *coli* or *V*. *cholerae*.

#### Exclusion criteria

The articles were excluded if they met any of the following conditions: **i**. published before 2013; **ii**. not written in English; **iii.** did not present original results such as reviews, editors, posters, seminar reports, or books; **iv.** describe microbiological contamination in aquaculture recirculation systems but not in aquaponic systems; **v.** focused on fungal as a probiotic; **vi.** not published in peer-reviewed scientific journals.

### Study selection process and data extraction

The Rayyan tool for systematic literature review (http://www.rayyan.ai/) was used to screen the articles. Two researchers (AAJ and LAV), independently performed the preliminary selection of the studies. First, duplicate articles were removed, and then, AAJ and LAV conducted a blind screening of the articles through the titles and abstracts. Only articles that met the inclusion criteria were included in the study, while those that did not met these criteria were excluded. The full text of the selected articles was reviewed independently by AAJ and LAV, who extracted the relevant data for qualitative analysis. Each researcher collated the following information: first author, year of publication, the objective of the study, study design, pathogenic microorganisms, section of contamination, and sources of contamination.

### Quality assessment

The quality and risk of bias of the studies were assessed using the Cochrane risk-of-bias tool in Cochrane Collaboration’s software Review Manager Version 5.4.1. This tool contains seven domains: (1) random sequence bias (selection bias), (2) allocation concealment (selection bias), (3) blinding of participants and personnel (performance bias), (4) blinding of outcomes assessment (detection bias), (5) incomplete outcomes data (attrition bias), (6) selective reporting (reporting bias), and (7) other bias [38]. Two reviewers (AAJ and LAV), assessed the quality of the articles and classified them as high quality, low quality, and unclear quality.

### Data synthesis

A narrative synthesis was conducted, providing a qualitative summary of the evidence by identifying patterns, trends, and relationships across the studies. The missing data were managed by assessing the quality and Risk of Bias of the included studies using the Cochrone domainsand by excluding studies that did not meet the eligibility criteria (S2 Table)

## Results

### Summary of the selection process

**Fig 1:**
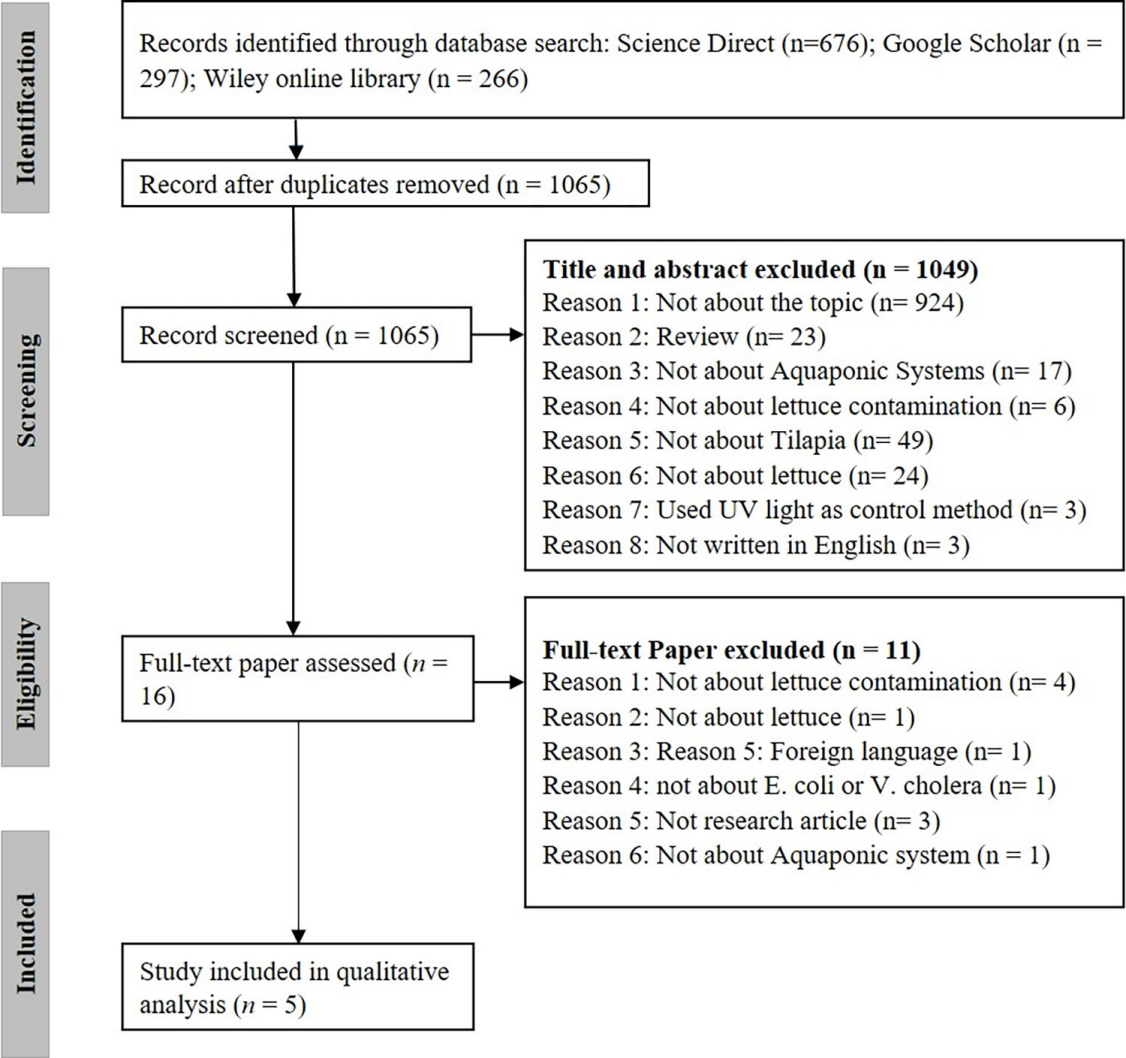
Preference Reporting Items for Systematic Review and Meta-Analysis (PRISMA) flowchart of the study selection process.

A total of 1,239 articles were retrieved from the three databases, using the search terms. 1,065 articles remained after the removal of duplicates (Fig 1). After screening these 1,065 articles by title and abstract, 1,049 articles were excluded because they did not meet the inclusion criteria. The reasons for exclusion were as follows: review articles (n = 23), not about aquaponic systems (n = 17), not about tilapia contamination (n = 6), not about lettuce (n = 24), not written in English (n = 3), not about the use of *Bacillus* strain as probiotics to control *E*. *coli* and *V*. *cholerae* (n = 3). Sixteen articles were selected for full-text review. However, eleven of these were excluded for the following reasons: not discussing lettuce contamination in aquaponic systems (n = 4), focusing on probiotics used to control microorganisms that are not *E*. *coli* and *V*. *cholera*e (n = 1), written in Portuguese (n = 1), describing contamination unrelated to lettuce (n = 1), not based on research studies (n = 3), and describing contamination but not in aquaponic (n = 1). Following full-text review, five articles were included in the systematic review for qualitative analysis (S1 Table). None of the 1,239 articles investigated the potential of lettuce being contaminated by pathogenic bacteria from tilapia and no included study reported the use of *Bacillus* strains as probiotics to control *E*. *coli* and *V*. *cholerae* in aquaponic systems.

### Quality assessment

**Fig 2:**
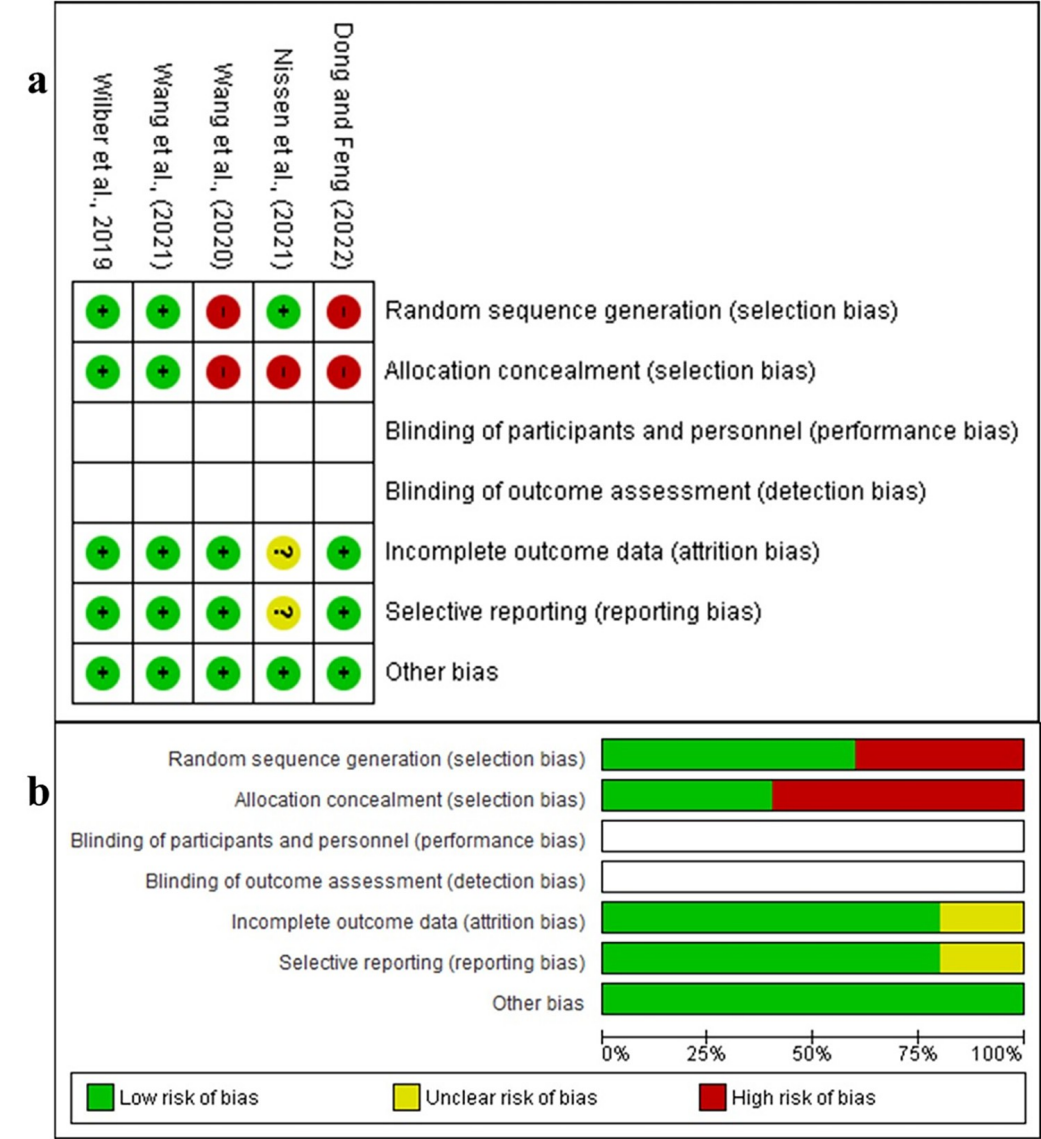
Quality assessment of the included studies: a) individual studies. b) Summary of risk of bias. The analysis was performed using the Cochrane risk-of-bias tool in Cochrane Collaboration’s software Review Manager Version 5.4.1.

The quality and risk of bias were evaluated using Review Manager Version 5.4.1. with the results shown in Fig 2. In the domain of random sequence generation (selection bias), two studies [39, 40], were identified as having a high risk of bias (40%), due to the lack of clarity regarding the randomization method. These studies did not clearly define the treatment or specify the components of each treatment contained. For the allocation concealment domain (selection bias), three included studies [39–41] were considered to have a higher risk of bias (60%), as they did not describe the allocation method, whether based on weight, age, or sex. Blinding of participant and personnel domain (performance bias), as well as blinding of outcome assessment (detection bias), was not applicable for any of the evaluated studies, since they are not clinical studies, and it is not possible to blind the participant even the personnel. Regarding the incomplete outcome data (attrition bias) and selective reporting (reporting bias), one included study [41] had an unclear risk of bias (25%) due to the insufficient data concerning the contamination source. Other bias refers to the study design and all studies (100%), were deemed a low risk of bias, as they adequately described the methodology used to achieve the results (S2 Table).

### Water quality and microbiological count

Two of the included studies reported the water quality parameters in aquaponic systems. These studies, mensured temperature (27.5±1.5°C; 22.9±0.1°C), pH (6.9±0.6; 6.5±0.0), dissolved oxygen (6.1±0.7mg/l; 7.8±0.03), electrical conductivity (1.1±0.4 ds/m; 1.52±0.04 ds/m), and nutrients availability (NH4^+^ 1.8±0.4, NO2^-^ 0.8±0.2, NO3^-^ 74.7±10.8). According to these studies, these parameters supported the growth of the *E*.*coli*.

For microbiological count, only three studies reported the Colony Forming Unit (CFU) of microorganisms in lettuce. In two of these studies, the CFU/g/ml count in lettuce leaves was 1.90 log10 and 2.3 log10 for Psychrotrophic bacteria; 3.46 log10 and 1.0 log10 for *Enterobacteriaceae*; 3.2 log 10 for aerobic mesophiles, and 1.93 log10 for coliforms. The other study reported a range of 3.7–4.0 log CFU/g of bacteria in lettuce from aquaponic systems.

### Microbiological contamination of lettuce in aquaponic systems

The data for the microbiological contamination analysis were extracted from five articles that studied the growth of lettuce alongside tilapia in aquaponic systems (Table 1). The results indicated that lettuce reared in aquaponic systems with tilapia can be contaminated by *Enterobacteriaceae*, *Coliforms*, *Aerobic mesophilic bacteria*, *Psychrotrophic bacteria*, *lactic acid bacteria*, *enterococci*, *yeast* and *molds*, *pseudomonas spp*, *Pseudomonas aeruginosa*, *Aeromonas hydrophila*, and *Shiga Toxin-producing E*. *coli* (STEC). None of the included studies reported contamination of Lettuce by *V*. *cholerae*.

**Table 1:** Section and source of the microbiological contamination in the aquaponic system.

Pathogenic microorganisms	Section of contamination	Source of contamination	Reference
*Enterobacteriaceae*; Coliforms; Aerobic mesophilic bacteria; *Psychrotrophic* bacteria; Lactic acid bacteria; *Enterococci*, *Pseudomonas spp*.; Yeasts and molds.	Leaves	Not Described	Nissen et al., (2021)
*Enterobacteriaceae*; Coliforms; Aerobic mesophilic bacteria; *Psychrotrophic* bacteria; Lactic acid bacteria; *Enterococci*; *Pseudomonas spp*.; Yeasts and molds.	lettuce roots		
*Enterobacteriaceae*; Coliforms; Aerobic mesophilic bactéria; Lactic acid bacteria; *Enterococci*; *Pseudomonas spp*.; Yeasts and molds.	Biofilter		
*Enterobacteriaceae*; Coliforms; Aerobic mesophilic bacteria; *Psychrotrophic* bacteria; Lactic acid bacteria; *Enterococci*; *Pseudomonas spp*.; Yeasts and molds.	Fishes		
*Enterobacteriaceae*; Coliforms; Aerobic mesophilic bacteria; *Psychrotrophic* bacteria; Lactic acid bacteria; *Pseudomonas spp*.; *Enterococci*; Yeasts and molds.	Fish tank		
*Enterobacteriaceae*; Coliforms; Aerobic mesophilic bacteria; Lactic acid bacteria; *Pseudomonas spp*.; *Enterococci*; Yeasts and molds,	Water		
*E*. *coli* (ASV1628),	Farm’s shoes	Open environment	Dong and Feng (2022)
*Pseudomonas aeruginosa*,	Farm’s shoes and lettuce	Not described	
*Aeromonas hydrophila*	Lettuce	Water	
Shiga-Toxin *Escherichia coli* (STEC)	Water, Fish feces, Lettuce roots surfaces, Internal leaves, Internal roots	Fish feces	Wang et al., (2021)
Shiga-Toxin *Escherichia coli* (STEC)	Water, Fish Feces, Lettuce roots Surface	Fish feces	Wang et al., (2020)
*Enterobacteria*; Aerobic mesophiles; *Psychrophilic bacteria*	Leaves	Water	Wilber et al., 2019

The results further show that microbiological contamination by various pathogenic bacteria can occur in both the roots and leaves of lettuce. The analysis revealed that the predominant pathogenic bacteria in leaves were *Enterobacteriaceae*, *coliforms*, and *Shiga Toxin-producing E*. *coli*. These pathogens were also detected in lettuce roots, tilapia, fish feces, water, and fish tanks. *Shiga Toxin-producing E*. *coli* was internalized into the lettuce leaves. Yeast and molds were the least abundant microorganisms. Although one included study did not specify which parts of lettuce were contaminated by pathogenic bacteria, it demonstrated that lettuce can be contaminated by *Aeromonas hydrophila* and *Pseudomonas aeruginosa* (S3 Table).

Regarding the source of lettuce contamination in aquaponic systems, 80% of the included studies mention water and fish feces as the primary sources of contamination. Therefore, these sources can be considered the main source of microbiological contamination in aquaponic systems. However, none of the included studies specifically mentioned tilapia rearing in aquaponic systems as a source of lettuce contamination. Moreover, no included study found in the searched databases reported instance of tilapia being challenged with *E*. *coli* or *V*. *cholerae* in aquaponic systems.

### Use of *Bacillus* strains as probiotics to control *E*. *coli* and *V*. *cholerae* in aquaponic systems

This systematic review considered the use of *B*. *subtilis*, and *B*. *licheniformis* as probiotics to control *E*. *coli* and *V*. *cholerae* as both species have been extensively used in aquaculture and have shown potential for controlling pathogenic bacteria. However, the studies included in this review did not report the use of these Bacillus species to control *E*. *coli* and *V*. *cholerae* in aquaponic systems.

## Discussion

This systematic review aimed to address two key research questions: first, to explore existing studies concerning lettuce contamination by pathogenic bacteria in aquaponic systems, and second, to assess the effectiveness of *B*. *subtilis* and *B*. *licheniformis* as probiotics in controlling *E*. *coli* and V. *cholerae* within such systems. To answer these questions, we critically evaluated five of the most relevant studies on the topic.

Overall, it appears that in aquaponic environments, the use of nutrient-rich water from fish tanks to grow plants increases the risk of contamination of edible parts of vegetables by pathogenic microorganisms. If the vegetables are consumed raw, a significant number of pathogenic bacteria can survive on the produce posing a public health hazard [42, 43].

In fish-rearing systems, factors such as the discharge of organic matter, environmental conditions, and untreated water can influence microbial metabolic processes and promote microbial proliferation [44–46]. According to [46], temperatures between 30–35°C, low levels of dissolved oxygen (<5mg/l), higher levels of NH3 (1-5mg/l), and pH (8.0) are associated with pathogens outbreaks in fish farms. The growth of *E*. *coli* in aquatic environments is influenced by various environmental factors, including temperature, pH, dissolved oxygen, nutrient availability, and humidity. This pathogen can survive for an extended period, up to 91 days, in aquatic environments at normal temperatures [47]. A study carried out by [48] reported that *E*. *coli* O177:H7 is resistant to temperature variation and can survive for 12 weeks in water at 25°C.

The isolation of pathogenic bacteria from foods is alarming because their presence poses a food safety risk, with potential implications for public health [49]. The presence of 100 CFU/100ml of coliforms in effluents, can significantly increase public health risk [42]. For instance, [50], reported the presence of 6.94 log10 CFU/g and 3.25 log10 CFU/g of aerobic mesophilic bacteria and coliforms, respectively in lettuce samples from organic farming systems. Similarly, [44], reported 7.57± 0.57 log10 CFU/g of aerobic mesophilic bacteria and below 3.38 log10 CFU/g of total coliforms in lettuce samples.

[51] investigated the microbial quality of lettuce leaves finding 5.3 ± 0.7 log CFU/g of aerobic mesophilic bacteria; 5.2±0.8 CFU/g of Enterobacteriaceae, and 4.3±0.7 CFU/g, of coliforms. [52] reported 2.87±0.76 x 10^9^ CFU/g of human pathogens bacteria in lettuce samples from different marketplaces. The number of Colony Forming Units per gram in lettuce samples was 6.35±0.69log10 CFU/g for aerobic mesophilic bacteria, 5.82±1.01log10 CFU/g for Psychotropic microorganisms, and 5.16±1.01 CFU/g for Enterobacteriaceae [53]. In the study of (49), the CFU/g of aerobic mesophilic bacteria and total coliforms were 4.82±2.55 log10 CFU/g and 3.50±1.00 CFU/g, respectively.

The presence of *E*. *coli* at around 10^5^ CFU/g or 1–3.8 CFU/g as well as coliforms at ≤4log 10 CFU/g or 3–6.4 CFU/g in food is a signal of public health risk [42, 54]. According to [55], bacterial count above 10^3^ CFU/g in food, exceeds the permissible limit recommended by the World Health Organization (WHO) and the International Commission on Microbiological Specification for Food (CMSF) standards.

The analysis of the reviewed studies revealed that *Enterobacteriaceae*, *coliforms*, and *Shiga-toxin-producing E*. *coli* play a significant role in the microbiological contamination of lettuce leaves in aquaponic systems. The *Enterobacteriaceae*, *E*. *coli*, and other microorganisms mentioned in the studies, such as *Aerobic mesophilic count*, *psychrotrophic microorganisms*, *Yeast and mold*, *lactic acid bacteria*, *A*. *hydrophila*, and *Pseudomonas spp* were found in lettuce grown in the organic farming system and also were isolated from tilapia faces [53, 56]. These pathogenic bacteria have been found in the effluent water, the root zone [57], and fish tanks [58]. *E*. *coli* can internalize in plants grown in hydroponic systems through the roots and move up to the edible parts of the plants. This internalization increases when roots are exposed to high densities of pathogens [59]. According to [60], enteric pathogens can survive outside their animal host and attach to plants by forming of biofilms, and internalization into plant tissue. The presence of those pathogens, specifically *Shiga toxin-producing E*. *coli*, in lettuce leaves is a public health concern [61]. Studies have shown that all samples from organic systems contain *E*. *coli* and *coliforms* [22, 62, 63].

The included studies did not report the presence of *V*. *cholerae* in aquaponic systems, likely, because none of them specifically investigated *V*. *cholerae*. However, studies targeting *V*. *cholerae* in tilapia and rearing water reported the isolation of this pathogen. In Taiwan, Bangladesh, and Egypt, [64–66], isolated *V*. *cholerae* from tilapia and from freshwater ponds in which tilapia was raised. Some researchers challenged tilapia with *V*. *cholerae* in closed systems and they reported colonization of tilapia by different strains of *V*. *cholerae* and its presence in rearing water. In Tanzania, [35] challenging tilapia with four strains of *V*. *cholerae* (*V*. *cholerae O1* Classical biotype, *V*. *cholerae O1*, EI Tor biotype, *V*. *cholerae O1* Δtox T, and *V*. *cholerae* no-O1), by inoculating it in the water. On days 1, 2, 3, 7, and 14, they observed that all strains of *V*. *cholerae* had colonized tilapia and these strains were also detected in aquarium water. In Egypt, [67, 68], conducted a study by injecting *V*. *cholerae* intraperitoneally into the tilapia reared in closed systems, and they reported that tilapia were colonized by *V*. *cholerae* after 10 days.

Regarding the source of contamination, the analysis of the included studies showed that water and fish feces are the main sources of contamination in aquaponic systems. Researchers have identified a link between aquaponic systems and the water source concerning microbial contamination [69]. Feces and water are common sources of pathogenic microorganisms in fresh produce [70]. In aquaponic systems, pathogens enter when proper hygiene practices are not applied [61]. Those pathogens can survive for extended periods in water [71]. Pathogenic microorganisms such as *Coliforms*, *Yeast* and *Mold*, *E*. *coli*, *Pseudomonas*, *A*. *hydrophila*, and *Enterobacteriaceae* have been detected in water samples from tilapia rearing system [20, 22, 43, 56]. If the water used in aquaponic systems is not treated, the microbial count of *Enterobacteriaceae*, *E*. *coli*, and *total bacteria* increases. The prevalence of these pathogens is influenced by favorable water temperatures [61]. According to [72], microbial density increases when the water temperature reaches approximately 21°C.

The included studies did not report the use of *B*. *subtilis* and *B*. *licheniformis* as probiotics to control *E*. *coli* and *V*. *cholerae*, however, these two Bacillus promote lettuce growth in aquaponic systems by regulating the microbiome [73] and improving weight gained, specific growth rate, feed conversion ratio, and thrombocyte counts in the bloodstream of Nile tilapia [27, 74].

These two probiotics have also been shown to reduce the Proteobacteria Phylum (to which *E*. *coli* belongs) in Nile tilapia cultured in aquaponic systems [27]. Their administration can enhance the immune systems of Nile tilapia by boosting their defense mechanisms [75]. *B*. *subtilis* has been shown to control vibriosis, *Aeromonas hydrophila*, and *Streptococcus aureus* in reared fish [76–78] and protects tilapia against bacterial infection in the ponds [79]. It can also inhibit the proliferation of pathogenic species of vibrio in *Letopenaeus Vannamei*, *Penaeus monodon*, and tilapia through exclusive competition mechanism in aquaculture [80, 81].

In the present review, we identified notable patterns of bias among the included studies. For example, 50% of the studies exhibited a high risk of bias in two domains: random sequence generation and allocation concealment. Additionally, 25% of the studies showed a high risk of bias in the domains of incomplete outcome data and selective reporting. The randomization and allocation methods were often unclear, raising concerns about the reliability of their findings. Moreover, the method used for detecting pathogenic bacteria in the study design labeled as another bias in this systematic review, was conventional, and probably failed to detect microorganisms that cannot thrive under such conditions. In one study, incomplete outcome data resulted from not reporting the source of contamination, casting doubt on the accuracy of its results and affecting our systematic review findings. Many of the studies analyzed, used both conventional and molecular methods for detect microorganisms in aquaponic systems. Overall, the high risk of bias across the studies reviewed stemmed from methodological disparities. A common limitation was the lack of a contamination analysis specifically targeting the lettuce leaves, the edible part of the plant. These methodological variations significantly impacted the outcomes of our systematic review.

## Conclusion

This systematic review revealed that roots and leaves of lettuce grown in aquaponic systems can be contaminated by pathogenic bacteria. The most common bacteria contaminating lettuce leaves in aquaponic systems include Enterobacteriaceae, Coliforms, and *Shiga Toxin-Producing E*. *coli*. In aquaponic systems, *Shiga-toxin producing E*. *coli* can internalize into lettuce leaves, and potentially infect humans when raw lettuce is consumed. *V*. *cholerae* is the causative agent of cholera, and commonly inhabits freshwater environments, with fish serving as its reservoir. Therefore, its presence in aquaponic systems must be monitored to ensure the safety of harvested vegetables. To improve pathogen identification and provide comprehensive insights into the microbial landscape in aquaponic systems, conventional methods should be combined with molecular techniques. Since water and fish feces are the main sources of pathogenic bacteria in these systems, further research is needed to explore the role of tilapia acting as a contamination source of vegetables grown in aquaponic systems and investigate the effectiveness of Bacillus strains in preventing human pathogenic bacteria.

## Supporting information

S1 Checklist PRISMA 2020 checklist. (DOCX)

S1 Protocol Protocol for systematic review already registered in Open Science Framework (OSF) with the associated project: osf.io/va3nk and registration Doi: https://doi.org/10.17605/OSF.IO/K64EN. (DOCX)

S1 Table Studies identified in the databases. (XLSX)

S2 Table Author judgement of Risk of Bias assessment. (DOCX)

S3 Table Data extracted from the primary research sources. (DOCX)
