# Supplementary material for: Microbiological contamination of lettuce (Lactuca sativa) reared with tilapia in aquaponic systems and use of bacillus strains as probiotics to prevent diseases: A systematic review
Source: PLoS One. 2024 Nov 11;19(11):e0313022. doi: 10.1371/journal.pone.0313022 (PMC11554229; doi:10.1371/journal.pone.0313022)
Supplement: S1 Protocol — (DOCX) [file pone.0313022.s002.docx]

**Contamination of Lettuce (*Lactuca sativa*) by Pathogenic Bacteria and Use of Bacillus as Probiotics to Control *Escherichia coli* and *Vibrio cholerae* in Aquaponic Systems: A Systematic Review**

^1^Angélica Adiação Jossefa*****, Sokoine University of Agriculture, Morogora, PO Box 3004, Tanzania: [angelica.jossefa@sacids.org](mailto:angelica.jossefa@sacids.org)

^2^Leonildo dos Anjo Viagem, Sokoine University of Agriculture, Morogoro, PO Box 3004, Tanzania: [leonildo.viagem@sacids.org](mailto:leonildo.viagem@sacids.org)

^3^Bruno da Silva Cerozi, University of São Paulo, Avenue Padua Dias, 11, PO Box 9, Piracicaba, São Paulo, 134118-900, Brazil: [brunno.cerozi@usp.br](mailto:brunno.cerozi@usp.br)

^4^Sebastian Wilson Chenyambuga, Sokoine University of Agriculture, Morogora, PO Box 3004, Tanzania: [chenya@sua.ac.tz](mailto:chenya@sua.ac.tz)

***Corresponding author:** [angelica.jossefa@sacids.org](mailto:angelica.jossefa@sacids.org)

**Abstract**

**Background:** Lettuce is the most cultured vegetable in aquaponic systems and is most commonly consumed as a raw vegetable. Lettuce is also one of the most common vegetables involved in foodborne outbreaks worldwide. Illnesses and deaths caused by the consumption of vegetables contaminated by pathogenic bacteria are a constant threat to public health worldwide. Among the pathogenic bacteria, *Escherichia coli* and *Vibrio cholera* are important pathogens that can circulate in the water and can infect humans. Previous studies recommend further research to evaluate microbiological contamination in aquaponic systems to guarantee the food safety of the products. Moreover, it is important to search for healthy methods to prevent foodborne pathogen contamination in aquaponic systems. One of the healthiest methods is the use of probiotics that can control pathogenic bacteria, making the products safe for human consumption. Bacillus strains have been used as probiotics in livestock and agriculture; however, there is limited information on the use of Bacillus strains as probiotics in aquaponic systems.

**Method:** A systematic review will include articles published from 2013 to 2023 and written in English. The following databases will be searched: Google Scholar, Science Direct, and the Wiley online library. To search the articles in these databases, search terms will be defined, and a search strategy will be used in each database connecting the search terms with the Booleans. To screen the articles after being exported into databases, the Rayyan tool will be used to find and eliminate duplicate articles. Then, two reviewers will screen the articles in the same program using the eligibility criteria previously defined. The full text of the selected articles will be read by two reviewers to extract the evidence of the studies that will be included in the analysis. The quality of the studies and risk of bias will be assessed using the Cochrane risk-of-bias tool in Cochrane Collaboration’s software Review Manager Version 5.4.

**Discussion:** This systematic review focuses on efforts to ensure the food safety of products from aquaponic systems through the design of cultivation methods that avoid microbiological contamination in aquaponic systems.

**Systematic review protocol registration**: This protocol has been registered at the Open Science Framework ([osf.io/va3nk](file:///D:\Systematic%20review\Systematic%20review%20journal\osf.io\va3nk)) with registration Doi: <https://doi.org/10.17605/OSF.IO/K64EN>.

**Keywords:** vegetables, pathogenic bacteria, tilapia, Probiotics.

**Background**

Aquaculture is a food production sector that grows more than the other food production sectors and is important to food and nutrition security (1–4). The sector has great potential to feed and nourish the world's growing population, and it is estimated that aquaculture production will provide an average of 21.4 kg per capita in 2030 (44). The rapid growth of aquaculture has negatively affected the environment, making the adoption of sustainable aquaculture necessary to meet the growing demand for animal food worldwide (44). To make aquaculture sustainable, many farmers around the world have adopted aquaponic systems, which are a form of a recirculation food production technology in which water is not exchanged (5). Aquaponic systems combine aquaculture and hydroponics in an integrated system where different organisms live symbiotically, providing benefits to each other (5). This food production system has been adopted almost all over the world due to its efficiency in food production and the guarantee of food safety (6,7). However, food production in aquaponic systems has raised many questions regarding the safety of vegetables grown (8) due to the risk of contamination of microorganisms from fish tanks (9).

According to (10,11), in aquaponic systems, microorganisms such as *Escherichia coli* and Vibrio cholerae can circulate within the system through the water and survive. Cultured fish can serve as reservoirs for disseminating pathogens to aquatic ecosystems (12,13). In aquaponic systems, many researchers, such as (10,14–19), have recommended carrying out more studies regarding the microbial contamination of vegetables because there are several paths for the introduction of pathogenic bacteria. Moreover, it is important to distinguish between beneficial bacteria and those that can cause diseases in humans. These authors reported that it is important to explore how and when microbial contamination occurs in aquaponic systems and called for additional studies due to worries about food safety and proposed research to be done on preventive strategies that can decrease the contamination risks of vegetables.

The most cultured species in aquaponic systems are lettuce (5) and Nile tilapia (20). Lettuce is the most common vegetable consumed raw, and it is the most common food associated with foodborne diseases worldwide (21). Nile tilapia contain diverse microbiota, such as *Streptococcus agalactiae*, *Flavobacterium columnare*, *Edwaedsiella tarda*, *Aeromonas salmonicida*, *Streptococcus iniae*, *Aeromonas sp*, *Citrobacter freundii*, *Escherichia coli*, *Plesiomonas shigelloides*, *Vibrio sp*, S*almonella*, *Staphylococcus* and *Bacillus* (22–24), some of which are pathogenic to humans, and their transmission can be by consumption of contaminated food (25,26). According to (9), growing contaminated fish in aquaponic systems can cause foodborne illness, and (26) identified foodborne pathogenic bacteria in fresh vegetables such as lettuce grown in aquaponic systems.

Due to the risks of microbiological contamination of vegetables grown in aquaponic systems, it is recommended to adopt prevention methods to control diseases (15). The use of probiotics may be a safe alternative for preventing diseases and ensuring quality food for consumers (27) because they do not pose a risk to the health of cultured fish and vegetables or humans (15). Probiotics are promoted due to the restriction on the use of antibiotics in animal feeds as growth promoters (27) and their health benefits, such as increasing the digestion and assimilation of nutrients, preventing the development of pathogens, improving environmental parameters, and balancing intestinal microbiota (28). In aquaponics, probiotics are used as an alternative to antibiotics (29), and the application of these microorganisms can be by supplementation in the feed or by inoculation in the water, and they can be applied individually or in combination (30)*.* The results found by (31) show that the use of *Bacillus subtilis* in Nile tilapia can improve the adaptative immune response through the modulation of intestinal microbiota. *B. subtilis* has no hazardous effects on the host, even to the consumer (27), and it is used as a probiotic in spore or vegetative form; both have high stability to the surroundings, heat, gastric conditions, and moisture (45). *B. subtilis* and *B. licheniformis* can improve growth performance and reduce pathogenic intestinal microbiota in Nile tilapia (32). According to (33), *B. subtilis* can be mixed with malic acid to reduce pathogens in the gut of Nile tilapia. *B. licheniformis* can be combined with other probiotics, giving good results in the host, and can be used in aquaculture (46). The beneficial effects of Bacillus include the modulation of intestinal microbiota, antimicrobial activity, and growth promotion (46).

This systematic review intends to assess the studies that have already been performed in aquaponic systems about the contamination of vegetables by pathogenic bacteria and identify the probiotics that are used to prevent disease in this food production system and document their efficacy.

**Method**

**Objective and research question**

This systematic review aims to provide a comprehensive analysis of contamination of lettuce grown in aquaponic systems and the potential use of Bacillus strains as probiotics to control E. coli and V. cholerae.

The research questions are as follows:

1. What is known about the microbiological contamination of lettuce cultivated together with tilapia in aquaponic systems?
2. What is known about the effect of *B. subtilis* and *B. licheniformis* as probiotics in controlling *E. coli* and *V. cholerae* in aquaponic systems?

**Design**

This protocol for systematic review was developed following the Preferred Reporting Items for Systematic Review and Meta-analysis Protocols (PRISMA-P) 2015 statement (34). The the additional file 1 shows the PRISMA checklist. The protocol was registered on Open Science Framework (OSF) with the associated project: [osf.io/va3nk](file:///D:\Systematic%20review\Systematic%20review%20journal\osf.io\va3nk) and registration Doi: <https://doi.org/10.17605/OSF.IO/K64EN>.

**Database Source and Search Strategy**

This systematic review will include articles published in the field of aquaculture with respect to microbial contamination of lettuce and the use of *B. subtilis* and *B. licheniformis* as probiotics to control *E. coli* and *V. cholerae* in aquaponic systems. The articles will be searched in the following databases: Google Scholar, Science Direct, and Wiley online library using the following search strategy: 1. “microbial” AND “contamination” AND “lettuce” AND “aquaponic system”; 2. “lettuce” AND “*Bacillus subtilis*” AND “E. coli” OR “Vibrio cholerae” AND “aquaponic systems”; 3. “lettuce” AND *“Bacillus licheniformis”* AND “E. coli” OR “V. cholerae” AND “Aquaponic system”. The relevant references that were cited in the included studies, but cannot be found in the databases using the search terms, will be searched manually and cross-checked to include them in the study.

**Eligibility criteria**

**Inclusion criteria**

To include the articles in the study, the following inclusion criteria will be used: **i**. articles from 2013 to 2023; **ii.** written in English language; **iii.** available in full-text; **iv**. with original research that combines Nile tilapia and lettuce farming in aquaponic systems; v. studies where Nile tilapia was challenged with *E. coli* or *V. cholerae*; **iv.** articles that target *B. subtilis* and *B. licheniformis* as a probiotic to control *E. coli* or *V. cholerae.*

**Exclusion criteria**

The exclusion criteria will be as follows: **i***.* articles published before 2013; **ii**. Articles that were not written in the English language; **iii.** Articles that do not present original results such as reviews, editors, posters, seminar reports, books, and without full text available; **iv.** articles that describe microbiological contamination in recirculation systems in aquaculture but not aquaponic systems; **v.** articles with fungi as a probiotic; **vi.** articles that were not published in peer-reviewed scientific journals will be excluded.

**Study Selection process**

For screening the articles, the Rayyan tool for systematic literature review (<http://www.rayyan.ai/>) will be used. Two researchers (AAJ and LAV) will perform the preliminary selection of the studies. First, one of the researchers will exclude duplicate articles, and then, two researchers (blindly) will screen the articles through titles and abstracts based on the inclusion and exclusion criteria. Only the articles with the inclusion criteria will be included, and the articles that will not respond to these criteria will be excluded. For the conflict that cannot be overcome by the two researchers, a third researcher will be invited to resolve the conflict. Full text for the included articles will be read by two researchers (blindly) to collect the relevant data that will be included in the quantitative analysis. Fig. 1 summarizes how the process of screening the articles was conducted.

**Fig. 1.** PRISMA diagram for article screening and selection.

**Data extraction**

In the included articles, two researchers (AAJ and LAV) will collect the relevant information for each study separately. This relevant information will be listed in a table that will be transformed to enable the analysis of the data (Table 1).

**Table 1. Information will be collected in each selected study.**

| First Author |  |
| --- | --- |
| Year of publication |  |
| Objective of the study |  |
| Study design |  |
| Pathogenic Microorganism |  |
| Contaminated section |  |
| Source of the contamination |  |

**Main outcome**

The main outcomes of this systematic review are:

1. Microbiological contamination in lettuce cultivated in aquaponic systems, including the contamination sources.
2. Efficacy of Bacillus strains as probiotics in controlling Escherichia coli and Vibrio cholerae in aquaponic systems

**Risk of bias assessment**

The quality and risk of bias will be assessed using the Cochrane risk-of-bias tool in Cochrane Collaboration’s software Review Manager Version 5.4.1. This tool contains seven domains: (1) random sequence bias (selection bias), (2) allocation concealment (selection bias), (3) blinding of participants and personnel (performance bias), (4) blinding of outcomes assessment (detection bias), (5) incomplete outcomes data (attrition bias), (6) selective reporting (reporting bias), and (7) other biases (47). The quality will be accessed by two reviewers **(**AAJ and LAV) following the following grades: high quality, low quality, and unclear quality.

**Discussion**

Aquaponics is a recirculation aquaculture system where plants, fish, and microorganisms live in dynamic equilibrium (35). Water circulating from fish tanks to plants in hydroponic systems without pretreatment can be a potential link to pathogenic microorganism infection through fish (48). According to (36,37), rearing contaminated fish in aquaponic systems can be a primary source of foodborne pathogens and can contaminate the systems with pathogenic microorganisms due to the ability of the pathogens to circulate into the system through the water (10). Similarly, water contaminated by pathogenic bacteria can be a link to contamination of the root surface in hydroponic systems(36,37). It is well known that lettuce grown in aquaponic systems can be contaminated by pathogenic bacteria from fish tanks and other sources in aquaponic systems, such as water, biofilters, and human activities. Contamination of lettuce in aquaponic systems can reduce productivity (38), and green leaves can be the source of foodborne illnesses in humans (39). However, in studies performed by (37) and (36), water contaminated with *E. col* did not cause the internalization of pathogenic bacteria within the roots and edible parts of lettuce in aquaponic systems, suggesting that there are some other sources of contamination. Additionally, in their studies, *E. coli* was not found in hydroponic systems, but it was present in water from fish tanks, probably due to the sanitization that was done before starting the culture, which may have avoided cross-contamination between the systems (36).

*Escherichia coli* is an Enterobacteriaceae that can be found in fish feces, water, and on the root surfaces of plants in aquaponic systems)37)). *Vibrio cholerae* is an important human pathogen that can proliferate in the aquatic environment and spread in the human intestine (40). According to (41), detecting *E. coli* on lettuce heads is possible after fertilizing water with manure. Proteobacteria, Actinobacteria, Cyanobacteria, Bacteroidetes, Firmicutes, and Pseudomonas are the most abundant genera found in aquaponic systems in which lettuce is cultured (48). (42) reported the presence of *Nitrospira*, *Terrimonas*, *Blastocatella*, *Sphingomonadaceae*, *Saprospiraceae*, *Luteolibacter*, *Burkholderiaceae*, and Flavobacterium in lettuce roots, biofilters, and sump samples in aquaponic systems.

*Bacillus subtilis* is an attractive probiotic in aquaculture production to control pathogenic bacteria in tilapia fish farming. Supplementation of this probiotic in Nile tilapia farms can improve tilapia growth due to its ability to populate the intestine of tilapia (49). Studies that used Bacillus in aquaculture report that this probiotic improves digestion and food assimilation and prevents disease onset (28). Studies carried out by Tachibana (2020) showed that the use of *B. subtilis* and *B. licheniformis* has positive effects on the intestinal microbiota of fish and zootechnical performance and can modify the intestinal microbiota by reducing pathogenic microorganisms, establishing a population of beneficial microorganisms in the host. It has been shown that using *B. subtilis* and *B. licheniformis* in Nile tilapia as probiotics reduces intestinal pathogenic bacteria and improves the growth of the fish (33,43). *Bacillus subtilis* was used in Nile tilapia challenged with *Streptococcus agalatiae* in aquaculture to prevent infection and reduce Nile tilapia mortality (31).

**Declaration**

**Ethics approval and consent of the participants**

Not applicable

**Consent of publication**

Not applicable

**Availability of data and material**

Not applicable

**Competing interests**

The authors declare that no competing interests exist.

**Funding**

The systematic review will be financed by the Partnership for Skills in Applied Sciences, Engineering, and Technology (PASET) through the Regional Scholarship and Innovation Fund (Rsif) awarded to AA Josefa and LA Viagem to carry out PhD studies at Sokoine University of Agriculture**.** The funders had no role in the study design, collection, and analysis of the information, the decision to publish, or any decision to publish the final manuscript related to the review.

**Acknowledgment**

Acknowledgments go to the Regional Scholarship and Innovation Fund (Rsif) for funding this systematic review and Sokoine University of Agriculture (SUA).

**Author contribution**

AAJ and LAV: Conceptualization, methodology, writing original draft; ET, BSC and SWC: Writing-review & editing and supervision. All the authors have read and approved the final manuscript.

**Author details**

^1.2^ PhD students in the Pos Graduate Program in Animal Science, Department of Animal, Aquiculture, and Range Sciences, Sokoine University of Agriculture, Morogora, PO Box 3004, Tanzania. ^3^ Assistant Professor at Department of Animal Science, College of Agriculture, University of Sao Paulo, Aveue Padua Dias, 11, PO Box 9, Piracicaba, São Paulo, 134118-900, Brazil. ^4^ Associate Professor in Animal Breeding and Genetics at Department of Animal, Aquaculture and Range Sciences, Sokoine University of Agriculture, PO Box 3004, Morogora, Tanzania.

**Supporting information**

**Additional file 1. PRISMA checklist**

**References**

1. Kibenge FS. Emerging viruses in aquaculture. Current Opinion in Virology. Elsevier B.V.; 2019; 34:97–103.

2. Goswami M, Shambhugowda YB, Sathiyanarayanan A, Pinto N, Duscher A, Ovissipour R, et al. Cellular Aquaculture: Prospects and Challenges. Micromachines. MDPI; 2022; 13:1-20.

3. Sabo-Attwood T, Apul OG, Bisesi JH, Kane AS, Saleh NB. Nanoscale applications in aquaculture: Opportunities for improved production and disease control. Journal of Fish Diseases. 2021; 44:359–370.

4. Subasinghe R, Soto D, Jia J. Global aquaculture and its role in sustainable development. Rev Aquac. 2009; 1:2–9.

5. Bailey DS, Ferrarezi RS. Valuation of vegetable crops produced in the UVI Commercial Aquaponic System. Aquac Rep. 2017; 7:77–82.

6. Monsees H, Suhl J, Paul M, Kloas W, Dannehl D, Würtz S. Lettuce (*Lactuca sativa*, variety Salanova) production in decoupled aquaponic systems: Same yield and similar quality as in conventional hydroponic systems but drastically reduced greenhouse gas emissions by saving inorganic fertilizer. PLoS One. 2019; 14:1-23.

7. Wirza R, Nazir S. Urban aquaponics farming and cities- a systematic literature review. Reviews on Environmental Health. 2021; 36:47–61.

8. Wielgosz ZJ, Anderson TS, Timmons MB. Microbial effects on the production of aquaponically grown lettuce. Horticulturae. 2017; 3:1-11.

9. Wang YJ, Deering AJ, Kim HJ. The occurrence of Shiga toxin-producing E. Coli in aquaponic and hydroponic systems. Horticulturae. 2020; 6:1-13.

10. Elumalai SD, Shaw AM, Pattillo DA, Currey CJ, Rosentrater KA, Xie K. Influence of UV treatment on the food safety status of a model aquaponic system. Water (Switzerland). 2017; 9:1-11.

11. Fox BK, Howerton R, Tamaru CS. Construction of Automatic Bell Siphons for Backyard Aquaponic Systems. Biotchnology. 2010; 10:1-11.

12. Halpern M, Izhaki I. Fish as hosts of Vibrio cholerae. Frontiers in Microbiology. Frontiers Research Foundation; 2017; 8:1-7.

13. Senderovich Y, Izhaki I, Halpern M. Fish as reservoirs and vectors of Vibrio cholerae. PLoS One. 2010; 5:1-5.

14. Goddek S, Joyce A, Kotzen B, Burnell Editors GM. Aquaponics Food Production Systems Combined Aquaculture and Hydroponic Production Technologies for the Future. 2019; p.619.

15. Rivas-García T, González-Estrada RR, Chiquito-Contreras RG, Reyes-Pérez JJ, González-Salas U, Hernández-Montiel LG, et al. Biocontrol of phytopathogens under aquaponics systems. Water (Switzerland). MDPI AG; 2020; 12:1-15.

16. Sawyer T. Food safety and E. Coli in aquaponic and hydroponic systems. Horticulturae. 2021; 7:1–5.

17. Hollyer J, Tamaru C, Riggs A, Klinger-Bowen R, Howerton R, Okimoto D, et al. On-Farm Food Safety: Aquaponics. Food Safety and Technology. 2009; 38:1-8.

18. Weller DL, Saylor L, Turkon P. Total coliform and generic E. Coli levels, and salmonella presence in eight experimental aquaponics and hydroponics systems: A brief report highlighting exploratory data. Horticulturae. 2020; 6:1–9.

19. Kasozi N, Kaiser H, Wilhelmi B. Determination of Phylloplane Associated Bacteria of Lettuce from a Small-Scale Aquaponic System via 16S rRNA Gene Amplicon Sequence Analysis. Horticulturae. 2022; 8:1-16.

20. Krastanova M, Sirakov I, Ivanova-Kirilova S, Yarkov D, Orozova P. Aquaponic systems: biological and technological parameters. Biotechnology and Biotechnological Equipment. Taylor and Francis Ltd.; 2022; 36:305–316.

21. Cardinale M, Grube M, Erlacher A, Quehenberger J, Berg G. Bacterial networks and co-occurrence relationships in the lettuce root microbiota. Environ Microbiol. 2015; 17:239–252.

22. Banerjee G, Ray AK. Bacterial symbiosis in the fish gut and its role in health and metabolism. Symbiosis. 2017; 29:1-11.

23. Bereded NK, Curto M, Domig KJ, Abebe GB, Fanta SW, Waidbacher H, et al. Metabarcoding analyses of gut microbiota of nile tilapia (Oreochromis niloticus) from lake awassa and lake chamo, ethiopia. Microorganisms. 2020; 8:1–19.

24. Hamom A, Alam MMM, Mahbub Iqbal M, Ibrahim Khalil SM q, Parven M, Sumon TA, et al. Identification of Pathogenic Bacteria from Diseased Nile Tilapia Oreochromis niloticus with their Sensitivity to Antibiotics. Int J Curr Microbiol Appl Sci. 2020; 9:1716–1738.

25. Bereded NK, Abebe GB, Fanta SW, Curto M, Waidbacher H, Meimberg H, et al. The gut bacterial microbiome of Nile tilapia (Oreochromis niloticus) from lakes across an altitudinal gradient. BMC Microbiol. 2022; 22:1-19.

26. Sawyer T. Food safety and E. Coli in aquaponic and hydroponic systems. Horticulturae. 2021; 7:1–5.

27. Ruiz Sella SRB, Bueno T, de Oliveira AAB, Karp SG, Soccol CR. *Bacillus subtilis* natto as a potential probiotic in animal nutrition. Critical Reviews in Biotechnology. Taylor and Francis Ltd.; 2021; 41:355–369.

28. Olmos J, Acosta M, Mendoza G, Pitones V. *Bacillus subtilis*, an ideal probiotic bacterium to shrimp and fish aquaculture that increase feed digestibility, prevent microbial diseases, and avoid water pollution. Archives of Microbiology. 2020, 202:427–435.

29. Haygood AM, Jha R. Strategies to modulate the intestinal microbiota of Tilapia (Oreochromis sp.) in aquaculture: a review. Reviews in Aquaculture. 2018, 10:320–333.

30. Hai N V. The use of probiotics in aquaculture. Journal of Applied Microbiology. 2015; 119:917–935.

31. Guimarães MC, Cerezo IM, Fernandez-Alarcon MF, Natori MM, Sato LY, Kato CAT, et al. Oral Administration of Probiotics (*Bacillus subtilis* and Lactobacillus plantarum) in Nile Tilapia (Oreochromis niloticus) Vaccinated and Challenged with *Streptococcus agalactiae*. Fishes. 2022; 7:1-18.

32. Tachibana L, Telli GS, Dias D de C, Gonçalves GS, Guimarães MC, Ishikawa CM, et al. *Bacillus subtilis* and *Bacillus licheniformis* in diets for Nile tilapia (Oreochromis niloticus): Effects on growth performance, gut microbiota modulation and innate immunology. Aquac Res. 2021; 52:1630–1642.

33. Hassaan MS, Soltan MA, Jarmołowicz S, Abdo HS. Combined effects of dietary malic acid and *Bacillus subtilis* on growth, gut microbiota and blood parameters of Nile tilapia (Oreochromis niloticus). Aquac Nutr. 2018; 24:83–93.

34. Moher D, Shamseer L, Clarke M, Ghersi D, Liberati A, Petticrew M, et al. Preferred reporting items for systematic review and meta-analysis protocols (PRISMA-P) 2015 statement. Revista Espanola de Nutricion Humana y Dietetica. 2016; 20:148–160.

35. Eck M, Sare AR, Massart S, Schmautz Z, Junge R, Smits THM, et al. Exploring bacterial communities in aquaponic systems. Water. 2019; 11:1-16.

36. Wang YJ. The Effects of Microbiomes on Food Crop Yield and Quality in Aquaponic Systems. PhD Thesis, Purdue University. 2021. P.221.

37. Wang YJ, Deering AJ, Kim HJ. The occurrence of Shiga toxin-producing E. Coli in aquaponic and hydroponic systems. Horticulturae. 2020; 6:1-13.

38. Day JA, Diener C, Otwell AE, Tams KE, Bebout B, Detweiler AM, et al. Lettuce (Lactuca sativa) productivity influenced by microbial inocula under nitrogen-limited conditions in aquaponics. PLoS One. 2021; 16:1-14.

39. Gómez-Aldapa CA, Rangel-Vargas E, Refugio Torres-Vitela MA, Villarruel-López A, Acevedo-Sandoval OA, Gordillo-Martínez AJ, et al. Antibacterial Activities of Hibiscus sabdariffa Extracts and Chemical Sanitizers Directly on Green Leaves Contaminated with Foodborne Pathogens. J Food Prot. 2018; 81:209–217.

40. Wang J, Yan M, Gao H, Lu X, Kan B. Vibrio cholerae Colonization of Soft-Shelled Turtles. Applied and Environmental Microbiology. 2017; 83:1-14.

41. Weller DL, Kovac J, Roof S, Kent DJ, Tokman JI, Kowalcyk B, et al. Survival of *Escherichia coli* on lettuce under field conditions encountered in the Northeastern United States. J Food Prot. 2017; 80:1214–221.

42. Eck M, Szekely I, Massart S, Jijakli MH. Microorganisms in aquaponics: Insights on the composition of the root microbiome of lettuces of varying age. In: Acta Horticulturae. International Society for Horticultural Science; 2021. p. 213–219.

43. Tachibana L, Telli GS, Dias D de C, Gonçalves GS, Guimarães MC, Ishikawa CM, et al. *Bacillus subtilis* and *Bacillus licheniformis* in diets for Nile tilapia (*Oreochromis niloticus*): Effects on growth performance, gut microbiota modulation and innate immunology. Aquac Res. 2021; 52:1630–1642.

44. FAO. The State of World Fisheries and Aquaculture 2022. (2022). In The State of World Fisheries and Aquaculture. [<https://doi.org/10.4060/cc0461en>] site visited on 10/4/2023.

45. Lee N-K, Kim W‒S, Paik H-D. Bacillus strains as human probiotics: characterization, safety, microbiome, and probiotic carrier. Food Sci Biotchnol. 2019; 28:1297-1305.

46. Ramirez-Olea H, Reyes-Ballesteros B, Chaves-Santoscoy RA. Potential application of the probiotic *Bacillus licheniformis* as an adjuvant in the treatment of diseases in human and animals: A systematic review. Frontier in Microbiology, 2022; doi:10.3389/fmicb.2022.993451.

47. Higgin JPT, Altman DG, Sterne JAC. Capter 8: Assessing risk of bias in included studies. In: Higgin JPT, Churchill R, Chandler J, Cumpston MS, Cochrane Handbook for Systematic Review of Interventions version 5.2.0 (updated June 2017), Cochrane, 2017. Available from [www.training.cochrane.org/handbook](http://www.training.cochrane.org/handbook).

48. Deng Mand Feng H. Microbial Community Analysis and Food Safety Practice Survey-Based Hazard Identification and Risk Assessment for Controlled. Frontiers in Microbiology, 2022; 13:1 – 15.

49. Galagarza OA, Smith SA, Drahos DJ, Eifert JD, William RC, Kuhn DD. Modulation of innate immunity in Nile tilapia (*Oreochromis niloticis*) by dietary supplementation of *Bacillus subtilis* endospores. Fish and Shelifish Immunology, 2018; 83: 171-179.
