# Supplementary material for: Microbiological contamination of lettuce (Lactuca sativa) reared with tilapia in aquaponic systems and use of bacillus strains as probiotics to prevent diseases: A systematic review
Source: PLoS One. 2024 Nov 11;19(11):e0313022. doi: 10.1371/journal.pone.0313022 (PMC11554229; doi:10.1371/journal.pone.0313022)
Supplement: S2 Table — (DOCX) [file pone.0313022.s004.docx]

**S3 Table**. Author Judgement of Risk of Bias Assessment

| **Study** | **Domain** | **Author judgment** | **Support judgement** |
| --- | --- | --- | --- |
| Nissen et al., (2021) | Random sequence generation (selection bias) | High | The method of randomization not reported |
|  | Allocation Concealment (selection bias) | High | Random allocation not reported |
|  | Blinding of participants and personnel (performance bias) | N/A |  |
|  | Blinding of outcome assessment (detection bias) | N/A |  |
|  | Incomplete outcome data (attrition bias) | Unclear | The data is recorded for most parameters, but the source was not reported |
|  | Selective reporting (reporting bias) | Unclear | All prespecified outcomes were not reported |
|  | Study design (other bias) | Low | Were reported |
| Dong et al., (2022) | Random sequence generation (selection bias) | High | The method of randomization not reported |
|  | Allocation Concealment (selection bias) | High | Random allocation not reported |
|  | Blinding of participants and personnel (performance bias) | N/A |  |
|  | Blinding of outcome assessment (detection bias) | N/A |  |
|  | Incomplete outcome data (attrition bias) | Low | The data is recorded for all parameters |
|  | Selective reporting (reporting bias) | Low | All prespecified outcomes were reported |
|  | Study design (other bias) | Low | Were reported |
| Wang et al., (2021) | Random sequence generation (selection bias) | Low | The randomization is reported |
|  | Allocation Concealment (selection bias) | Low | Rondom allocation (Allocated by weight, and treatment) |
|  | Blinding of participants and personnel (performance bias) | N/A |  |
|  | Blinding of outcome assessment (detection bias) | N/A |  |
|  | Incomplete outcome data (attrition bias) | N/A | The data is recorded for all parameters |
|  | Selective reporting (reporting bias) | Low | All prespecified outcomes were reported |
|  | Study design (other bias) | Low | Were reported |
|  |  |  |  |

**S3 Table**. Continued

| **Study** | **Domain** | **Author judgment** | **Support judgement** |
| --- | --- | --- | --- |
| Wang et al., (2020) | Random sequence generation (selection bias) | Low | The method of randomization not reported |
|  | Allocation Concealment (selection bias) | Low | Random allocation not reported |
|  | Blinding of participants and personnel (performance bias) | N/A |  |
|  | Blinding of outcome assessment (detection bias) | N/A |  |
|  | Incomplete outcome data (attrition bias) | Low | The data is recorded for all parameters |
|  | Selective reporting (reporting bias) | Low | All prespecified outcomes were reported |
|  | Study design (other bias) | Low | Were reported |
| Wilber et al., 2019 | Random sequence generation (selection bias) | Low | The method of randomization not reported |
|  | Allocation Concealment (selection bias) | Low | Random allocation not reported |
|  | Blinding of participants and personnel (performance bias) | N/A |  |
|  | Blinding of outcome assessment (detection bias) | N/A |  |
|  | Incomplete outcome data (attrition bias) | Low | The data is recorded for all parameters |
|  | Selective reporting (reporting bias) | Low | All prespecified outcomes were reported |
|  | Study design (other bias) | Low | Were reported |
