# Supplementary material for: Microbiological contamination of lettuce (Lactuca sativa) reared with tilapia in aquaponic systems and use of bacillus strains as probiotics to prevent diseases: A systematic review
Source: PLoS One. 2024 Nov 11;19(11):e0313022. doi: 10.1371/journal.pone.0313022 (PMC11554229; doi:10.1371/journal.pone.0313022)
Supplement: S3 Table — (DOCX) [file pone.0313022.s005.docx]

**S4 Table**.  Data extracted from the primary research sources

| **Data extractors** | **Date of data extraction** | **Eligible criteria to include the study in the review** | **Data extracted from each study** | | | |
| --- | --- | --- | --- | --- | --- | --- |
|  |  |  | **Pathogenic microorganisms** | **Section of contamination** | **Source of contamination** | **Reference** |
| Angélica Adiação Jossefa/Leonildo dos Anjo Viagem | September, 2023 | 1. published between 2013 and 2023; 2**.** written in English; 3**.** available in full text; 4. contains original research that combines tilapia and lettuce farming in aquaponic systems; 5**.** Articles where tilapia was challenged with *E. coli* or *V. cholerae*; 6**.** focus on *B. subtilis* and *B. licheniformis* as a probiotic to control *E. coli* or *V. cholerae.* | *Enterobacteriaceae*; Coliforms; Aerobic mesophilic bacteria; *Psychrotrophic* bacteria; Lactic acid bacteria; *Enterococci*, *Pseudomonas spp*.; Yeasts and molds. | Leaves | Not Described | Nissen et al., (2021) |
| Angélica Adiação Jossefa/Leonildo dos Anjo Viagem | September, 2023 | 1. published between 2013 and 2023; 2**.** written in English; 3**.** available in full text; 4. contains original research that combines tilapia and lettuce farming in aquaponic systems; 5**.** Articles where tilapia was challenged with *E. coli* or *V. cholerae*; 6**.** focus on *B. subtilis* and *B. licheniformis* as a probiotic to control *E. coli* or *V. cholerae.* | *Enterobacteriaceae*; Coliforms; Aerobic mesophilic bacteria; *Psychrotrophic* bacteria; Lactic acid bacteria; *Enterococci*; *Pseudomonas spp.*; Yeasts and molds. | lettuce roots |  |  |
| Angélica Adiação Jossefa/Leonildo dos Anjo Viagem | September, 2023 | 1. published between 2013 and 2023; 2**.** written in English; 3**.** available in full text; 4. contain original research that combines tilapia and lettuce farming in aquaponic systems; 5**.** Articles where tilapia was challenged with *E. coli* or *V. cholerae*; 6**.** focus on *B. subtilis* and *B. licheniformis* as a probiotic to control *E. coli* or *V. cholerae.* | *Enterobacteriaceae*; Coliforms; Aerobic mesophilic bactéria; Lactic acid bacteria;  *Enterococci*; *Pseudomonas spp*.; Yeasts and molds. | Biofilter |  |  |
| Angélica Adiação Jossefa/Leonildo dos Anjo Viagem | September, 2023 | 1. published between 2013 and 2023; 2**.** written in English; 3**.** available in full text; 4. contain original research that combines tilapia and lettuce farming in aquaponic systems; 5**.** Articles where tilapia was challenged with *E. coli* or *V. cholerae*; 6**.** focus on *B. subtilis* and *B. licheniformis* as a probiotic to control *E. coli* or *V. cholerae.* | *Enterobacteriaceae*; Coliforms;  Aerobic mesophilic bacteria; *Psychrotrophic* bacteria; Lactic acid bacteria; *Enterococci*; *Pseudomonas spp*.;  Yeasts and molds. | Fishes |  |  |

**S4 Table**.  Continued

| **Data extractors** | **Date of data extraction** | **Eligible criteria to include the study in the review** | **Data extracted from each study** | | | |
| --- | --- | --- | --- | --- | --- | --- |
|  |  |  | **Pathogenic microorganisms** | **Section of contamination** | **Source of contamination** | **Reference** |
| Angélica Adiação Jossefa/Leonildo dos Anjo Viagem | September, 2023 | 1. published between 2013 and 2023; 2**.** written in English; 3**.** available in full text; 4. contain original research that combines tilapia and lettuce farming in aquaponic systems; 5**.** Articles where tilapia was challenged with *E. coli* or *V. cholerae*; 6**.** focus on *B. subtilis* and *B. licheniformis* as a probiotic to control *E. coli* or *V. cholerae.* | *Enterobacteriaceae*; Coliforms;  Aerobic mesophilic bacteria; *Psychrotrophic* bacteria; Lactic acid bacteria; *Pseudomonas spp*.;  *Enterococci*; Yeasts and molds. | Fish tank |  |  |
| Angélica Adiação Jossefa/Leonildo dos Anjo Viagem | September, 2023 | 1. published between 2013 and 2023; 2**.** written in English; 3**.** available in full text; 4. contain original research that combines tilapia and lettuce farming in aquaponic systems; 5**.** Articles where tilapia was challenged with *E. coli* or *V. cholerae*; 6**.** focus on *B. subtilis* and *B. licheniformis* as a probiotic to control *E. coli* or *V. cholerae.* | *Enterobacteriaceae*; Coliforms; Aerobic mesophilic bacteria; Lactic acid bacteria;  *Pseudomonas spp*.; *Enterococci*; Yeasts and molds, | Water |  |  |
| Angélica Adiação Jossefa/Leonildo dos Anjo Viagem | September, 2023 | 1. published between 2013 and 2023; 2**.** written in English; 3**.** available in full text; 4. contain original research that combines tilapia and lettuce farming in aquaponic systems; 5**.** Articles where tilapia was challenged with *E. coli* or *V. cholerae*; 6**.** focus on *B. subtilis* and *B. licheniformis* as a probiotic to control *E. coli* or *V. cholerae.* | *E. coli* (ASV1628), | Farm's shoes | Open environment | Dong and Feng (2022) |
| Angélica Adiação Jossefa/Leonildo dos Anjo Viagem | September, 2023 | 1. published between 2013 and 2023; 2**.** written in English; 3**.** available in full text; 4. contain original research that combines tilapia and lettuce farming in aquaponic systems; 5**.** Articles where tilapia was challenged with *E. coli* or *V. cholerae*; 6**.** focus on *B. subtilis* and *B. licheniformis* as a probiotic to control *E. coli* or *V. cholerae.* | *Pseudomonas aeruginosa*, | Farm's shoes and lettuce | Not described |  |

**S4 Table**.  Continued

| **Data extractors** | **Date of data extraction** | **Eligible criteria to include the study in the review** | **Data extracted from each study** | | | |
| --- | --- | --- | --- | --- | --- | --- |
|  |  |  | **Pathogenic microorganisms** | **Section of contamination** | **Source of contamination** | **Reference** |
| Angélica Adiação Jossefa/Leonildo dos Anjo Viagem | September, 2023 | 1. published between 2013 and 2023; 2**.** written in English; 3**.** available in full text; 4. contain original research that combines tilapia and lettuce farming in aquaponic systems; 5**.** Articles where tilapia was challenged with *E. coli* or *V. cholerae*; 6**.** focus on *B. subtilis* and *B. licheniformis* as a probiotic to control *E. coli* or *V. cholerae.* | *Aeromonas hydrophila* | Lettuce | Water |  |
| Angélica Adiação Jossefa/Leonildo dos Anjo Viagem | September, 2023 | 1. published between 2013 and 2023; 2**.** written in English; 3**.** available in full text; 4. contains original research that combines tilapia and lettuce farming in aquaponic systems; 5**.** Articles where tilapia was challenged with *E. coli* or *V. cholerae*; 6**.** focus on *B. subtilis* and *B. licheniformis* as a probiotic to control *E. coli* or *V. cholerae.* | Shiga-Toxin *Escherichia coli* (STEC) | Water (AS), Fish feces (AS), Lettuce roots surfaces (AS), Internal leaves (AS), Internal roots (AS) | Fish feces | Wang et al., (2021) |
| Angélica Adiação Jossefa/Leonildo dos Anjo Viagem | September, 2023 | 1. published between 2013 and 2023; 2**.** written in English; 3**.** available in full text; 4. contains original research that combines tilapia and lettuce farming in aquaponic systems; 5**.** Articles where tilapia was challenged with *E. coli* or *V. cholerae*; 6**.** focus on *B. subtilis* and *B. licheniformis* as a probiotic to control *E. coli* or *V. cholerae.* | Shiga-Toxin *Escherichia coli* (STEC) | Water, Fish Feces, Lettuce roots Surface | Fish feces | Wang et al., (2020) |
| Angélica Adiação Jossefa/Leonildo dos Anjo Viagem | September, 2023 | 1. published between 2013 and 2023; 2**.** written in English; 3**.** available in full text; 4. contains original research that combines tilapia and lettuce farming in aquaponic systems; 5**.** Articles where tilapia was challenged with *E. coli* or *V. cholerae*; 6**.** focus on *B. subtilis* and *B. licheniformis* as a probiotic to control *E. coli* or *V. cholerae.* | *Enterobacteria*; Aerobic mesophiles;  *Psychrophilic bacteria* | Leaves | Water | Wilber et al., 2019 |
